# Recommendations for Face Coverings While Exercising During the COVID-19 Pandemic

**DOI:** 10.1186/s40798-021-00309-7

**Published:** 2021-03-15

**Authors:** Jonathan Shurlock, Borja Muniz-Pardos, Ross Tucker, Norbert Bachl, Theodora Papadopoulou, Graham Holloway, Nigel Jones, Xavier Bigard, Karin Vonbank, David Niederseer, Joachim Meyer, Dennis Nowak, Andre Debruyne, Petra Zupet, Herbert Löllgen, Juergen M. Steinacker, Bernd Wolfarth, James L. J. Bilzon, Anca Ionescu, Michiko Dohi, Jeroen Swart, Demitri Constantinou, Victoriya Badtieva, Irina Zelenkova, Maurizio Casasco, Michael Geistlinger, Chiara Fossati, Federica Fagnani, Luigi Di Luigi, Nick Webborn, Konstantinos Angeloudis, Fergus M. Guppy, Patrick Singleton, Mike Miller, Fabio Pigozzi, Yannis P. Pitsiladis

**Affiliations:** 1 Somerset NHS Foundation Trust, Taunton, UK; 2 GENUD (Growth, Exercise, Nutrition and Development) research group, University of Zaragoza, Zaragoza, Spain; 3 World Rugby, Dublin, Ireland; 4 European Federation of Sports Medicine Associations (EFSMA), Lausanne, Switzerland; 5 International Federation of Sports Medicine (FIMS), Lausanne, Switzerland; 6 Institute of Sports Science, University of Vienna, Vienna, Austria; 7 Austrian Institute of Sports Medicine, Vienna, Austria; 8 British Association Sport and Exercise Medicine, Doncaster, UK; 9 Defence Medical Rehabilitation Centre (DMRC), Loughborough, UK; 10 British Cycling and University of Liverpool, Liverpool, UK; 11 Union Cycliste Internationale (UCI), Aigle, Switzerland; 12 Department of Pneumology, Pulmonary Function Laboratory, Medicine Clinic (KIMII), University of Vienna, Vienna, Austria; 13 Department of Cardiology, University Hospital Zurich, University Heart Centre, University of Zurich, Zurich, Switzerland; 14 German Respiratory Society (DGP), Berlin, Germany; 15 Lung Center Bogenhausen-Harlaching, Munich Clinic, Munich, Germany; 16 LMU Klinikum, Institute and Clinic for Occupational, Social and Environmental Medicine, Comprehensive Pneumology Center, member DZL, German Center for Lung Research, Munich, Germany; 17 Division of Sports and Rehabilitation Medicine, Ulm University Hospital, Ulm, Germany; 18 Department of Sports Medicine, Humboldt University and Charité University School of Medicine, Berlin, Germany; 19 Department for Health, University of Bath, Bath, UK; 20 Sport Medical Center, Japan Institute of Sports Sciences, Tokyo, Japan; 21 UCT Research Unit for Exercise Science and Sports Medicine, Cape Town, South Africa; 22 Centre for Exercise Science and Sports Medicine, University of the Witwatersrand, Johannesburg, South Africa; 23 I.M. Sechenov First Moscow State Medical University (Sechenov University), Ministry of Health of Russia, Moscow, Russian Federation; 24 Moscow Research and Practical Centre for Medical Rehabilitation, Restorative and Sports Medicine, Moscow Healthcare Department, Moscow, Russian Federation; 25 Italian Federation of Sports Medicine (FMSI), Rome, Italy; 26 Unit International Law, Department of Constitutional, International and European Law, University of Salzburg, Salzburg, Austria; 27 University of Rome “Foro Italico”, Rome, Italy; 28 Villa Stuart Sport Clinic, FIFA Medical Center of Excellence, Rome, Italy; 29 School of Sport and Service Management, University of Brighton, Eastbourne, UK; 30 Centre for Exercise Sciences and Sports Medicine, FIMS Collaborating Centre of Sports Medicine, Rome, Italy; 31 Centre for Stress and Age-related Disease, University of Brighton, Brighton, UK; 32 World Olympians Association, Lausanne, Switzerland

**Keywords:** SARS-CoV-2, COVID-19, mask, face covering, exercise

## Abstract

In an effort to reduce transmission and number of infections of the severe acute respiratory syndrome coronavirus 2 (SARS-CoV-2 or COVID-19) virus, governments and official bodies around the world have produced guidelines on the use of face masks and face coverings. While there is a growing body of recommendations for healthcare professionals and the wider population to use facial protection in “enclosed spaces” where minimal distancing from other individuals is not possible, there is a dearth of clear guidelines for individuals undertaking exercise and sporting activity. The present viewpoint aims to propose recommendations for face coverings while exercising during the COVID-19 pandemic that consider physical distancing, the environment, the density of active cases associated with the specific sports activity, and the practical use of face coverings in order to reduce potential viral transmission. Recommendations are provided on the basis of very limited available evidence in conjunction with the extensive collective clinical experience of the authors and acknowledging the need to consider the likelihood of the presence of the SARS-CoV-2 in the general population. We recommend that face coverings should be used in any environment considered to be of a high or moderate transmission risk, where tolerated and after individual risk assessment. In addition, as national caseloads fluctuate, individual sporting bodies should consider up to date guidance on the use of face coverings during sport and exercise, alongside other preventative measures.

## Key Points

- There are limited specific data available for the use of face coverings while exercising in the context of COVID-19.
- Decisions for the type of face covering used cannot be made in isolation and require consideration of additional risk factors including environment and local case rates.
- Where appropriate, decisions should be guided and supported by relevant sporting bodies or organizations.

## Introduction

In an effort to reduce transmission and number of infections of the severe acute respiratory syndrome coronavirus 2 (SARS-CoV-2) virus, governments and official bodies around the world have produced guidelines on the use of face masks (i.e., professionally manufactured masks, such as the ones used in healthcare) and face coverings (i.e., any type of fabric or material to cover one’s face) [1, 2]. In the UK, the Independent Scientific Advisory Group for Emergencies (SAGE) recently launched a comprehensive campaign to promote effective wearing of face coverings in enclosed public indoor spaces where minimal distancing from others is not possible [3]. This guidance to use facial protection in “enclosed spaces” where minimal distancing from other individuals is not possible is consistent with guidance from the World Health Organization (WHO) [4]. While there is a growing body of recommendations for healthcare professionals and the wider population, there is a dearth of clear guidelines for individuals undertaking exercise and sporting activity. A recent commentary hypothesized the risks and benefits of wearing face coverings while exercising [5], including current public beliefs that wearing face coverings during exercise will be accompanied by carbon dioxide (CO_2_) accumulation [5]. The present viewpoint aims to propose recommendations for face coverings while exercising during the COVID-19 pandemic that consider physical distancing, the environment, the density of active cases associated with the specific sports activity, and the practical use of face coverings in order to reduce potential viral transmission.

## Face Covering Rationale

The theoretical basis for the use of masks and other forms of face covering in the context of the SARS-CoV-2 pandemic is continuously being developed and updated. The present consensus is that transmission primarily occurs through the ejection of virus particles from the respiratory tract of an infected individual which is then absorbed through the eyes, nose, or mouth of the exposed person [6]. An increasing body of research also describes aerosolized transmission which may not only travel significant distances from the source (up to 6–9 m [7]) but may also remain suspended in the air for significant periods [8], especially in the absence of air turbulence. Droplet size seems a crucial factor, with large droplets falling through the air more quickly than they evaporate and landing within 1–2 m range, and small droplets evaporating more quickly than they fall and traveling beyond 2 m in the air [7]. A systematic review of case clusters found a majority of them were associated with indoor settings [9], though many of these clusters are reported from China during winter, when individuals were more likely to be indoors. Additional risk factors that have not been extensively examined are situations where the spread of droplets is increased due to a higher ventilation rate [10] and/or the drafting effect of these droplets in the air, as can occur during certain forms of exercise (e.g., running or cycling) [11]. A recent systematic review and meta-analysis of observational data suggests that the use of face coverings provides protection against infection for the general population, with an additional benefit afforded by the use of eye protection [12]. The authors concede however that face masks or face coverings alone cannot provide complete protection from infection and highlight the need for their use alongside other preventative practices such as physical distancing [12]. Varying degrees of protection are seen across the range of face coverings, from cloth style coverings to filtering face piece level 2 (FFP2) respirators [13]. While respirator masks have been shown to be most effective in reducing aerosolized exposure, across all mask types, the reduced risk appears to be relatively stable independent of sustained activity or duration of wear [13].

## Drafting and the External Environment

Aerodynamic studies suggest an increased potential range of droplet transfer from one individual to another during exertional performance [14] due to increased ventilation and respiratory volumes with increased exertion. Observational data measuring athlete respiratory volumes during maximal exertion suggest an optimal safe distance of 10–20 m when considering droplet transmission [15]. In their model, safety distance varied proportionally to exercise intensity, and therefore to the respiratory flow (e.g., 2 m at rest or 11.6 m when ventilation is 160 L/min) [15]. Direct measurement of droplet dispersion was not performed in either study [14, 15] but instead estimated on the basis of variations in respiratory flow [15] and without taking into account air turbulence during movement outdoors or the impact of the wind. Therefore, these studies do not sufficiently clarify the risk of contamination and spread of the coronavirus during sporting activities in the field, either by aerosols or by droplets. Despite the importance of high respiratory rate during exercise on the dispersion of droplets, there are no reliable data to date to support recommending safe distances during sporting activities. This will require the role played by air turbulence but also the effect of wind on the dispersion of droplets and aerosols to be clarified, especially during high-speed sports such as cycling.

The aforementioned models of viral transmission highlight the impact of various climatic conditions such as ambient temperature, wind, altitude and ultra-violet (UV) exposure. High ambient temperatures would be expected to increase sweat production and therefore shorten the duration of tolerable single face covering usage. High levels of humidity are likely to have a significant impact on an individual’s ability or willingness to wear a face covering while exercising and also appear to have a role in reducing viral transmission [16]. Notably, the choice of covering will profoundly impact heart rate, thermal stress, and subjective perception of discomfort due to the vastly different thermal properties and micro-climates created within the different masks [17]. An increased retention of water vapor and sweat within the mask might also affect the facial seal of the mask, furthermore increasing breathing resistance and potentially increasing the risk of transmission to the wearer via a wicking mechanism [18].

## Current Evidence and Recommendations with a Focus on Sport and Exercise

There is limited available evidence assessing the impact of face coverings on sporting performance. Face masks designed specifically to restrict air flow have been shown to negatively impact ratings of perceived exertion without a beneficial metabolic response during resistance training [19, 20]. Both surgical masks and N95/FFP2 respirator masks have been shown in a small (*N* = 12) study to limit ventilation, cardiopulmonary exercise capacity, and comfort in healthy active individuals performing incremental cycling tests until voluntary exhaustion [21]. In contrast, other work shows that the use of surgical masks during exercise (i.e., 1 h at low-moderate intensity) does not produce significant physiological effects or impairments in subjective perception [22]. There is no verified support for any undesirable effects of wearing masks or other forms of face covering during exercise in healthy individuals, despite recent non-peer reviewed lay press reports suggesting that the use of a face mask during exercise can be health-threatening due to hypercapnia (i.e., excessive CO_2_ re-breathing) and hypoxemia (i.e., decrease in the partial pressure of oxygen in the blood) [23, 24]. A recent report has suggested these unwanted side-effects are unlikely due to negligible amounts of CO_2_ being re-breathed and the lack of a hypoxic stimulus [25], although empirical evidence is required to confirm this. There are currently no experimental data on the impact of face coverings and different mask materials and thicknesses on sports performance in elite athletes.

The following recommendations are provided considering the potential risks of viral transmission associated with the activity being undertaken and the impact of face coverings on exercise performance. We propose three levels for risk of transmission—high, moderate, and low. The assignment of sporting activities to these levels is based on our assessment of three factors that are known to contribute to overall transmission risk and are incorporated into our proposed stratification in the following sequential manner. First, a characteristic identified as the physical distancing factor, which is the degree to which a sport exposes its participants to a risk of exposure to the virus. This is a function of the number of participants, their relative proximity (or potential proximity during performance) and the duration for which close proximity occurs. As per the WHO [4], a close exposure requires proximity for 15 min, used as a guide to assess this variable. Any activity that prevents physical distancing is considered to have a high transmission risk. Activity which allows physical distancing but continues to involve multiple participants in close proximity is considered to have moderate transmission risk, based on meta-analysis demonstrating a strong association between risk of infection and proximity to source [12]. Second, any environmental characteristic that contributes significantly to transmission risk was identified. In particular, being outdoors significantly reduces the risk of transmission [26], such that for any physical distancing characteristics, transmission risk is downgraded one level if the sport is performed outdoors (e.g., from *high* to *moderate*). The remainder of the above discussed environmental factors such as ambient temperature should be applied to individual decisions due to the likely impact on tolerability of wear, perception of discomfort, and efficacy of covering used. Third, an incidence characteristic, which refers to the density of new cases within a community, was applied. Where the assessment of case incidence can be guaranteed as a result of trustworthy comprehensive testing and screening, the risk of exposure to an active case and thus overall transmission is greatly reduced. As such, we apply a reduction in transmission risk by one level in these cases. In this regard, the degree to which a sports organization, government or local legislative body can provide data on active cases, or act to screen and test participants regularly to identify active cases, will contribute to reduced risk of exposure and disease spread.

Various examples of such incidence-based risk assessments exist [27–29]. Table 1, adapted from the risk mitigation procedures used by the World Rowing Federation [28], identifies a threshold of 50 cases per 100,000 population in the last seven days as indicative of *high risk*. Recent (7 days) cases between 20 and 50 per 100,000 constitute *medium risk*, while fewer than 20 cases per 100,000 indicate *low risk* of exposure. Individual sports may consider their own thresholds for identification of exposure risk, at the direction of government and public health organizations, in order to apply a risk reduction factor if desired.

**Table 1:** Risk attribution according to different regional scenarios of SARS-CoV-2

Risk	Region/county: new cases	Minimum recommended distance (m)	Sport activity	Weight room	Ergometer exercise
High	More than 50 cases per 100,000 in 7 days	1.5	Alone	Closed	Alone
Medium	Fewer than 50 cases per 100,000 in 7 days	1.5	Groups with safety avoiding drafting during exercise	Maximum capacity based on room size Disinfection Enhanced cleaning	1.5 m distance between ergometers Good ventilation
Low	Fewer than 20 cases per 100,000 in 7 days	1.5	Groups with safety	Maximum capacity based on room size Disinfection Hygiene	Good ventilation
No	Not applicable	No regulation	Groups	Open Disinfection Hygiene	Open

However, the incidence characteristic should be applied with caution, since these statistics may not always be accurately available, and nor may nationwide data apply to smaller regions where small outbreaks are occurring, or where no cases may exist. Furthermore, false negative results, the lag period between exposure to the virus and symptom appearance, and the relatively high proportion of asymptomatic cases, may underestimate the number of active cases when testing and screening are relied upon to perform this “filter” role. Therefore, in the absence of reliable and timely data, it may be prudent that individuals/teams/organizations assume a high risk of significant local transmission rates and therefore high risk of exposure.

**Fig. 1:**
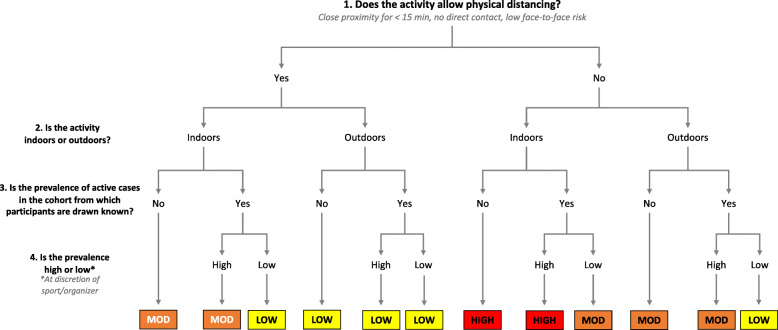
A flow chart of the risk assignment process of activities

In summary, the process followed to assign activities to risk levels is to firstly identify the physical distancing characteristic and then to perform a subjective assessment of participant proximity and duration. For outdoor activities, the risk level is reduced one level, and a second potential reduction in risk is available if a sport or regional legislator can defend a low incidence of active cases. In the absence of either outdoor participation, or an incidence characteristic that allows a reduction, the risk level is determined by the physical distancing characteristic and is either high for activities that prevent physical distancing, or moderate for sports that enable physical distancing. A flow chart of the process is summarized in Fig. 1.

### High Risk of Transmission

Indoor and group activities particularly where the nature of the activities does not allow adequate physical distancing, resulting in direct, face-to-face contact and/or prolonged exposure to other participants (e.g., basketball, rugby, football, volleyball, indoor gyms, team sport training sessions). Wearing a highly protective face covering (e.g., medical mask or N95) is recommended due to stronger association with protection from infection than single layered masks [12]. In sports where direct contact is an intended part of the game, such as rugby, adherence to this is unlikely to be practical. In such settings, decisions regarding face coverings should be taken in the context of additional measures to reduce risk.

### Moderate Risk of Transmission

Indoor activities with ≤ 2 individuals where appropriate distancing measures are possible (e.g., racquet sports) or outdoor activities where the nature of the activities limits the degree to which adequate physical distancing can occur and/or where drafting may occur (e.g., baseball, track events, peloton cycling, rugby, football, running in a crowded place). Wearing a minimum of a medium-thickness face covering (e.g., double layer cloth mask) is recommended [12, 30, 31].

### Low Risk of Transmission

Individual outdoor activities or group outdoor activities which allow for appropriate physical distancing measures and no possibility of drafting (e.g., individual running, singles tennis, golf). If physical distancing is possible throughout the entire period of exercise, sports without face covering is possible. When this is not feasible, wearing low-thickness face covering (e.g., single layer cloth face covering) is recommended [32].

Additionally, in those cases where a low prevalence is guaranteed and at the discretion of the sporting body or government tasked with regulation of the activity, the activity is further downgraded in risk, as shown in Fig. 1. Where there is convincing evidence of complete local suppression, i.e., no community transmission, a case-by-case assessment can be made for reverting to routine conditions without the use of face coverings.

### Unknown Risk of Transmission

In the context of the global transmission, viral genetic drift and regular genome sequencing the discovery of new variants of SARS-CoV-2 is inevitable. In the presence of limited available data, it may not be possible to draw conclusions about possible increased transmissibility, and caution should therefore be exercised with regard to risk assessment. If there is evidence or suspicion of local presence of variants with increased transmission such as the UK Variant Under Investigation (VUI-202012/01) [33], the situation should be treated as high risk until further evidence is available.

## Individual Exceptions

Face coverings are not well tolerated by all individuals especially those with pre-existing respiratory conditions that may prohibit prolonged usage. There is limited available evidence of the tolerability of face coverings by individuals with such conditions. Individuals with chronic obstructive pulmonary disease (COPD) and high dyspnea scale scores are less likely to tolerate the N95 mask. In these individuals with significant disease burden, a variety of respiratory parameters can be negatively impacted by N95 mask use [34]. Individuals with underlying respiratory conditions should undergo a physician-lead risk assessment, provided by the responsible organization in the professional and semi-professional setting, or with a relevant medical practitioner in the recreational setting. Furthermore, specific populations with disabilities may not be able to wear any form of face covering. Individuals suffering from severe anxiety, claustrophobia, or post-traumatic stress disorder may also feel unable to stay calm or function while wearing a face covering. Similarly, individuals with autism are extremely sensitive to touch and texture [35], which might cause sensory overload, panic, and/or anxiety. Notably, the team/organization should be responsible for performing this assessment where possible and should promote other alternative methods of transmission reduction for individuals with disabilities such as the use of full-face shields or scarfs instead of face masks, adequate physical distancing, and good hand hygiene. This would avoid undesirable situations such as the recent report revealing how a man with chronic asthma and serious breathing issues was forced to wear a face covering during a 1-h airplane journey [36]. The aforementioned recommendations and risk stratification levels should be adapted to the individual’s own risk profile and circumstances. These alternative methods imply changing policies, practices, and procedures, if needed, to accommodate individuals with a disability who are unable to wear a variety or all forms of face covering. Finally, an exception may be necessary for individuals in contact and combat sports that involve direct physical contact, which makes the wearing of masks impractical and significantly less effective.

The mandatory use of face covering includes individuals of different age ranges depending on national rules. In the UK for example, children under the age of 11 years are not required to wear face coverings [37], while the government of Spain declared the use of face covering as mandatory from the age of 6 years and also recommended their use in children between the ages of 3 and 5 years [38]. Other organizations have taken a more flexible and situation dependent position, encouraging the use of face coverings in all children over the age of 2 years when in public. They further recommend that this should only be done if it can be done in compliance with Centers for Disease Control and Prevention (CDC) guidance [39] avoiding frequently touching or removing the face covering [40].

## Face Covering Utility and Disposal

There is a lack of conclusive evidence on the optimum duration of single face covering usage. Decisions on when to discard or change a face covering should consider personal comfort, transmission risk of current environment, and accessibility to clean face coverings. Evidence does show a risk of contamination of the outside of masks with respiratory pathogens [41]. The emphasis must be on safe disposal of single use face coverings or storage and cleaning of reusable coverings.

Where an individual is participating in sporting activity under the umbrella of a team or club, the organization should provide safe disposal facilities for used face coverings. Clinical waste bins should be strategically placed at likely points of mask removal such as changing room exits, mirroring transition points from high to low-risk areas in hospitals. Organizations should also provide appropriate training to their members regarding safe removal and disposal of face masks. While these interventions inevitably entail a financial burden, it is anticipated that this is less than the cost of temporarily shutting down operations in the event of a localized outbreak.

Recreationally active individuals should dispose of their face masks in a sealed container or bag. Based on viral surface survival studies, the contained masks can then be disposed of in normal household waste after 4–7 days [42], although some have branded this approach as excessively cautious [43]. Where reusable face coverings are utilized, these should be washed after each use following appropriate washing recommendations (e.g., CDC recommendations [44]).

## General Recommendations

Guidance from all relevant authorities such as considering the mode of transport to training and using a face covering if traveling via public transport should be followed. Face covering should be used in enclosed spaces associated with sporting activities. Examples include group changing rooms or in a tunnel with two teams prior to outdoor performance. It is reasonable to remove face coverings to eat and drink and as far as is possible, such consumption should be done in outdoor spaces.

## Conclusion

The above recommendations are provided based on the limited available evidence in conjunction with the extensive collective clinical experience of the authors and acknowledging the need to consider the likelihood of the presence of the SARS-CoV-2 in the general population. As national caseloads fluctuate, individual sporting bodies should consider up to date guidance on the use of face coverings during sport and exercise, alongside other preventative measures. In summary, face coverings should be used in any environment considered to be of a high or moderate transmission risk, if tolerated and after individual risk assessment.

## Data Availability

Not applicable.

## Abbreviations

SAGE: Independent Scientific Advisory Group for Emergencies
WHO: World Health Organization
CO_2_: Carbon dioxide
UV: Ultraviolet
COPD: Chronic obstructive pulmonary disease
CDC: Centers for Disease Control and Prevention
